# Guideline Concordant Therapy Prolongs Survival in HER2-Positive Breast Cancer Patients: Results from a Large Population-Based Cohort of a Cancer Registry

**DOI:** 10.1155/2014/137304

**Published:** 2014-03-20

**Authors:** E. C. Inwald, O. Ortmann, F. Zeman, M. Koller, F. Hofstädter, M. Gerstenhauer, M. Klinkhammer-Schalke

**Affiliations:** ^1^Department of Gynecology and Obstetrics, University Medical Center Regensburg, Landshuter Straße 65, 93053 Regensburg, Germany; ^2^Center for Clinical Studies, University Hospital Regensburg, 93053 Regensburg, Germany; ^3^Institute of Pathology, University of Regensburg, 93053 Regensburg, Germany; ^4^Tumor Center Regensburg e.V., University of Regensburg, 93053 Regensburg, Germany

## Abstract

Even though randomized controlled clinical trials demonstrated improved survival by adjuvant trastuzumab treatment of HER2-positive breast cancer patients, data on its effect in clinical routine are scarce. This study evaluated the use and efficacy of trastuzumab in routine treatment of HER2-positive breast cancer patients. Data from the clinical cancer registry Regensburg (Germany) were analyzed. The present study investigated 6,991 female patients with primary invasive breast cancer. In premenopausal HER2-positive patients a considerable increase of trastuzumab therapy was observed from 58.1% in 2006 to 90.9% in 2011, whereas in postmenopausal patients trastuzumab was rather used on a constant rate of 49.1%. Best overall survival (OS) was found in HER2/steroid hormone receptor-positive patients receiving guideline concordant treatment with trastuzumab plus chemotherapy (CHT) plus antihormone therapy (AHT) with a 7-year OS rate of 96% compared to the non-trastuzumab group with a 7-year OS rate of 92%. In multivariable analysis, HER2-positive patients treated with CHT or AHT who did not get trastuzumab, had a worse 7-year OS (65%, *P* = 0.006 versus 79%, *P* = 0.017) than the control groups. This population-based study demonstrated that guideline concordant use of adjuvant trastuzumab improves OS for HER2-positive breast cancer patients treated in routine clinical care.

## 1. Introduction

One of the pivotal advancements in breast cancer research was the identification of HER2 overexpression as a significant predictor of both disease-free survival (DFS) and overall survival (OS) in breast cancer patients by Slamon et al. in 1987 [1]. HER2, a member of the epidermal growth factor receptor family of tyrosine kinases, is involved in cell growth and proliferation [2]. Overexpression and/or amplification of HER2 occurs in 15–25% of breast cancers and is associated with an unfavorable course of disease [1]. The development of trastuzumab has improved treatment results of HER2-positive breast cancer. Trastuzumab is a recombinant humanized monoclonal antibody directed against the extracellular domain of the transmembrane HER2 receptor [3]. Initially, the safety and efficacy of trastuzumab were evaluated in patients with HER2-positive metastatic breast cancer [4–8]. Trastuzumab was FDA-approved for treatment of metastatic breast cancer patients in 1998 [9] and in 2000 it was approved in Europe.

Later, five of six large phase III trials including more than 14,000 patients with HER2-positive early breast cancer demonstrated its efficacy in the adjuvant setting [10–15]. The joint analysis of the North American trials NSABP B-31 and NCCTG N9831 found that the addition of trastuzumab to chemotherapy resulted in a significant benefit in terms of DFS and OS for women with HER2-positive breast cancer [13]. After 3 years, the rate of DFS was 87.1% in patients receiving trastuzumab plus chemotherapy compared with 75.4% in patients in the standard chemotherapy arm (absolute difference 11.8 percentage points, HR 0.48, 95% CI 0.39–0.59; *P* < 0.0001). Regarding OS, also a benefit of trastuzumab plus chemotherapy was shown (94.3% in the combination therapy group versus 91.7% in the standard therapy group, absolute difference 2.5 percentage points) [13]. In the Herceptin Adjuvant Trial (HERA), after a median follow-up of 2 years, a significant absolute advantage in OS of 2.7% in the trastuzumab group over the non-trastuzumab control group was shown (92.4% versus 89.7%, HR 0.66, 95% CI 0.47–0.91; *P* = 0.0115) [11]. A second interim analysis of the BCIRG 006 study demonstrated superior DFS and OS in the trastuzumab arms after a median follow-up of 36 months [16]. The DFS was 83% and the OS 92% in patients receiving doxorubicin and cyclophosphamide followed by docetaxel and trastuzumab, in comparison with 77% and 86% in the control patients (HR 0.61 for DFS; *P* < 0.0001) [16]. At a median follow-up of 38 months, the DFS in the FinHER trial was longer with trastuzumab plus chemotherapy than with chemotherapy alone (89% versus 78%, HR 0.42, 95% CI 0.21–0.83; *P* < 0.01) [12]. Also OS was better in the trastuzumab group (96.3% versus 89.7%), with a reduction of the risk of dying (HR 0.41, 95% CI 0.16–1.08; *P* = 0.07) [12].

In summary, these studies showed that one year of trastuzumab treatment in combination with or sequentially after chemotherapy improved the relative risk for DFS by approximately 50% and OS by 30% irrespective of tumor size, nodal status, and type of chemotherapy. As a consequence, in 2006 trastuzumab was approved for adjuvant treatment of breast cancer in Europe.

Trastuzumab was the first FDA-approved monoclonal antibody targeting solid tumors [17]. Commonly, trastuzumab is well tolerated. Cardiac toxicity is considered to be the most serious adverse effect, but in the large trials it was almost always reversible [18, 19]. Meanwhile novel anti-HER2 strategies are under development like the combination of two HER2-targeted agents with nonoverlapping mechanisms of action to optimize HER2-directed therapy [20–22]. One potential approach is the combination of trastuzumab with the HER2 dimerization inhibitor pertuzumab [23, 24].

The positive results of controlled clinical trials notwithstanding, data on the effect of trastuzumab in clinical routine are scarce [25–29]. Thus, it is essential to ensure that treatment strategies, which are recommended in current national and international guidelines, are implemented in routine clinical care. As early as 1993 a systematic review suggested a positive impact of clinical guidelines on both the process and outcomes of care for several health conditions [30]. Nevertheless, it is difficult to demonstrate effects of treatment improvements on survival due to the lack of high quality clinical cancer registries with long term follow-up. Therefore, the intention of this population-based study was to evaluate guideline concordant treatment of HER2-positive breast cancer patients in the routine clinical adjuvant setting in a large cohort of more than 6,000 patients by analyzing data from a population-based regional cancer registry.

## 2. Material and Methods

### 2.1. Database

Data from the Tumor Centre Regensburg (Bavaria, Germany), a high quality population-based regional cancer registry covering a population of more than 2.2 million people of the districts of Upper Palatinate and Lower Bavaria, were analyzed. The clinical cancer registry Regensburg was founded in 1991 and currently disposes the follow-up of 240,655 patients. This cancer registry achieves a cross-sectorial documentation of all breast cancer patients in the area, following a rigorous prospective protocol. Documentation includes diagnosis, course of disease, therapies, and long term follow-up. Patient data stem from 53 regional hospitals, the University Hospital Regensburg, and more than 1,000 practicing doctors. The population-based data were routinely analyzed in each case and documented in the cancer registry on the basis of medical reports, pathology, and follow-up records. Mortality data were obtained from all regional registry offices.

### 2.2. Inclusion and Exclusion Criteria

**Figure 1:**
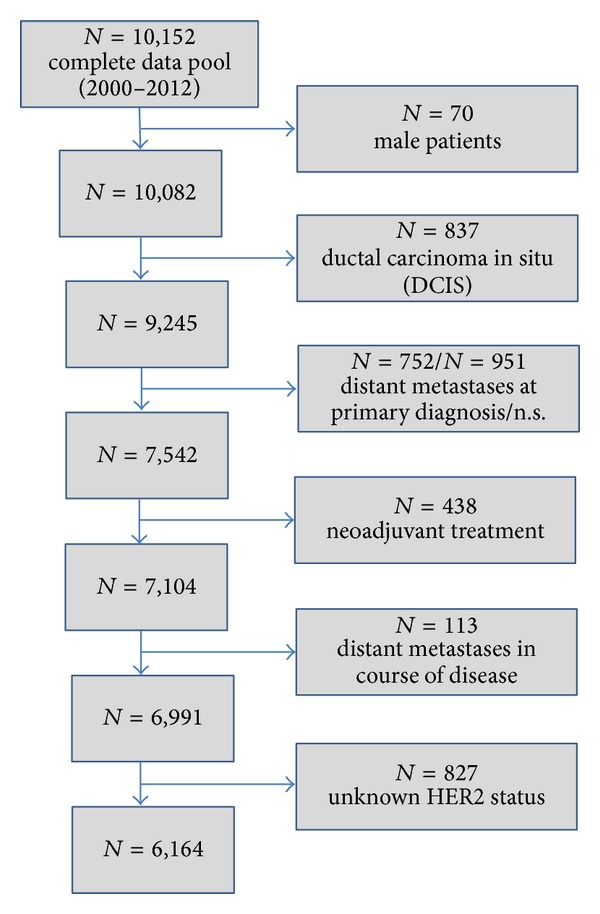
Scheme of data extraction.

The present analysis includes all female patients of the registry with primary, nonmetastatic (M0) invasive breast cancer diagnosed between January 2000 and December 2012 (13 years). Patients were followed up until May 2013. Exclusion criteria were male patients, ductal carcinoma in situ (DCIS), neoadjuvant treatment, and distant metastases at primary diagnosis or during the course of disease (Figure 1).

### 2.3. Analysis of HER2 Expression

HER2 testing was performed according to the German interdisciplinary S3 Guidelines for Diagnosis, Treatment and Follow-up Care of Breast Cancer (registry number 032-045OL of Association of the Scientific Medical Societies, AWMF) [31, 32] and to ASCO/CAP recommendations [33]. HER2-protein overexpression was analyzed by means of DAKO HercepTest, a semiquantitative immunohistochemical assay.

A total of six institutes of pathology were involved in these assessments. Consistency and quality control are ensured through biannual quality assurance conferences and the participation in round robin tests [34].

### 2.4. Primary Trastuzumab Therapy

In the trastuzumab group (1 year of trastuzumab with 3 weeks intervals of application) either concurrent or sequential administration relative to chemotherapy was performed. Different types of anthracycline- and taxane-based chemotherapy regimens were used according to the previously reported large controlled clinical trials [18].

### 2.5. Statistical Methods

Continuous data are expressed as means ± standard deviation (SD) and categorical data as frequency counts (percentages). Baseline characteristics of patients were compared between HER2 status by Student's *t*-test for continuous variables and by Pearson's chi-square tests for categorical variables. OS was calculated from the date of cancer diagnosis to the date of death from any cause. Patients who are not dead or patients without follow-up were classified as censored. To assess the influence of trastuzumab, chemotherapy (CHT), and antihormone therapy (AHT) on OS in HER2-positive patients and of CHT and AHT in HER2-negative patients, two multivariable Cox regression models were calculated. Both models were adjusted for the known confounding variables age, tumor size, nodal status, grading, receptor status, and Ki-67. Kaplan-Meier plots were used for graphical illustrations. For all combinations of trastuzumab, CHT, and AHT, odds ratios and corresponding 95% confidence intervals (95% CI) were calculated. All reported *P* values are two-sided, and a *P* value of 0.05 was considered the threshold of statistical significance. Calculations were made with the software packages SPSS 21.0.0.1 (Chicago, EUA) and R (version 2.14.2).

## 3. Results

### 3.1. Patient Characteristics and Histopathological Parameters

From the total data pool of breast tumor patients, 6,991 female patients with invasive early breast cancer according to the ICD-10 classification (C50) were extracted for further analysis (Figure 1). In 88.2% (6,164 patients) the HER2 status was available. In 11.8% (827 patients) the HER2 status was not available owing to missing information in the medical reports or no assessment. In the year 2000, the HER2 status was analyzed only in 16.3% of patients (70/430). This number increased to 84.2% of patients (383/455) in 2002 right up to 99.6% of patients (538/540) in 2012 (Table 1).

Only patients with known HER status were considered for further evaluation. Thus, a total of 6,164 patients with invasive breast cancer were taken into consideration for subsequent statistical analyses. Of these, 1,387 patients (22.5%) were premenopausal and 4,777 patients (77.5%) were postmenopausal. The mean age was 61 years (median: 62 years, range: 21–97 years). 5,030 patients (81.6%) were HER2 negative, whereas 1,134 patients (18.4%) were HER2 positive (overexpression and/or amplification). High grade tumors were more likely to be HER2 positive than low grade tumors. 44.1% (*n* = 500) of HER2-positive patients had poorly differentiated tumors, whereas only 6.2% (*n* = 70) were low grade. Estrogen receptor (ER) and progesterone receptor (PR) negative tumors were more likely to be HER2-positive than ER-positive and PR-positive tumors (23.9% versus 58.7%). Vice versa, in HER2-negative tumors 79.2% were ER and PR positive whereas only 10.7% were ER and PR negative. In HER2-positive tumors, lymphatic and vascular invasion was more frequent than in HER2-negative tumors (37.9% and 8.5% versus 26.6% and 5.2%). Low Ki-67 values ≤ 15% were predominant in HER2-negative tumors (45.8%) whereas higher Ki-67 categories (Ki-67 >25%) were found in HER2-positive patients.

### 3.2. HER2 Overexpression and Trastuzumab Therapy

Up to 2004, trastuzumab therapy was hardly administered in adjuvant treatment. Only about 2% of HER2-positive patients received trastuzumab from 2000 to 2004. Since trastuzumab approval for adjuvant therapy, the number of HER2-positive patients receiving the antibody continuously increased from 38.2% in 2006 to 66.2% in 2011. The analyses of age-related subcategories (pre- and postmenopausal patients) showed that nonuse of trastuzumab was found predominantly in postmenopausal women. Differences in trastuzumab therapy between premenopausal and postmenopausal patients were further analyzed (Table 2). There was a considerable increase of trastuzumab therapy in premenopausal patients from 58.1% in 2006 to 90.9% in 2011. The decrease of trastuzumab use in 2012 can be explained by incomplete documentation. In contrast, in postmenopausal patients trastuzumab was rather used on a constant rate of 49.1% from 2006 to 2011 after a first augmentation from 30.4% in 2006 to 53.8% in 2007. In the total population of HER2-positive patients, who were diagnosed between 2006 and 2012 (*n* = 660), only 52.7% (348 patients) received the appropriate treatment with trastuzumab. Among these, more premenopausal HER2-positive patients received trastuzumab in comparison to postmenopausal patients (67.2% (*n* = 119) premenopausal patients versus 47.4% (*n* = 229) postmenopausal patients).

To elucidate reasons for the insufficient application of trastuzumab, we further analyzed HER2-positive patients who did not receive trastuzumab between 2006 and 2012 with respect to their concomitant diseases. 312 HER2-positive patients did not receive trastuzumab. Out of these, 237 patients (76.0%) had at least one serious concomitant disease. 21 patients (6.7%) had no comorbidity and in 54 patients (17.3%) concomitant diseases were not documented. The majority of patients (58.2%) suffered from cardiopulmonary disease. Others had metabolic (12.2%), mental (8.9%), and gastrointestinal/hepatic/renal (4.2%) disorders (Table 3).

Furthermore, combinations of systemic therapies were analyzed since 2006, also contemplating CHT and AHT (Table 4). 34.3% (*n* = 221) of HER2-positive patients were treated with trastuzumab plus CHT plus AHT. 17.4% (*n* = 112) received trastuzumab plus CHT; 21.4% (*n* = 138) received only AHT. A considerable number of HER2-positive patients (15.0%, *n* = 97) received neither trastuzumab nor CHT nor AHT.

### 3.3. Survival Analyses

**Figure 2:**
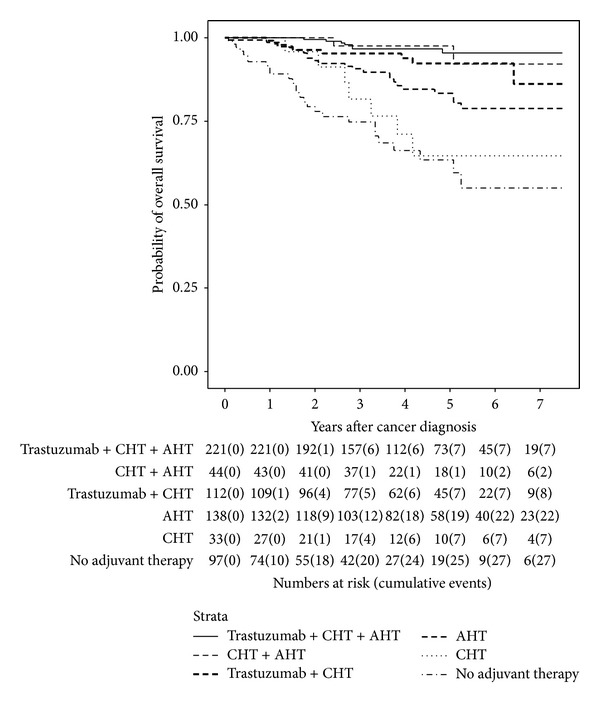
Kaplan-Meier plot of overall survival in years of HER2-positive patients based on adjuvant therapy.

**Figure 3:**
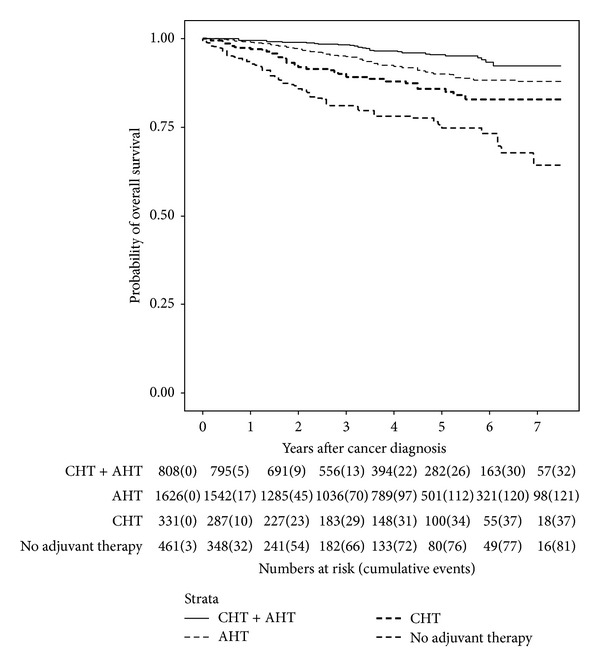
Kaplan-Meier plot of overall survival in years of HER2-negative patients based on adjuvant therapy.

Since patients received a number of different systemic treatments, analyses of trastuzumab effects were performed by comparing survival data obtained from these treatment groups (Table 5). HER2-positive patients who did not receive trastuzumab had a worse OS in all of the compared groups. Kaplan-Meier curves (Figures 2 and 3) show that guideline concordant therapy—that is, trastuzumab in HER2-positive, CHT in risk factor positive (high grade, large, nodal positive tumors, etc.), and AHT in ER/PR-positive patients—resulted in best survival rates. Deviation from guideline concordance reduced OS significantly. Best OS was found in HER2/ER/PR-positive patients receiving trastuzumab plus CHT plus AHT with a 7-year OS rate of 96%. Depriving HER2/ER/PR-positive patients of trastuzumab, 7-year OS rate deteriorated to 92%. Similarly, the nonuse of trastuzumab in HER2-positive/ER/PR-negative patients decreased 7-year OS rates from 86% to 65%. The worst OS of all patients was found in HER2-positive patients who received neither trastuzumab nor CHT nor AHT with a 7-year OS rate of 55%. Similarly, the impact of guideline concordant therapy was seen in HER2-negative patients whereas the effects were not that distinct. Consequently, an appreciable benefit of following guidelines was demonstrated. Remarkably, the addition of trastuzumab overcame the primarily worse outcome of HER2-positive patients indicating its efficacy. A particularly striking effect was found when comparing HER2-positive patients receiving no adjuvant therapy with triple-negative breast cancer patients receiving no adjuvant therapy at all. HER2-positive patients had an even worse survival (55%) than triple-negative breast cancer patients (64%).

By using Cox regression models (Tables 6(a) and 6(b)), it was demonstrated that HER2-positive patients (*n* = 516, 53 events) without trastuzumab/CHT/AHT therapy (*n* = 80, 21 events) showed the worst OS of all patients (HR = 10.44, 95% CI 3.02–36.06, *P* < 0.001) compared to patients receiving trastuzumab/CHT/AHT therapy (reference group). Also patients without trastuzumab therapy but only CHT (HR = 9.50, 95%-CI 1.90–47.43, *P* = 0.006) or AHT (HR = 4.28, 95%-CI 1.30–14.05, *P* = 0.017), respectively, had worse survival than the control groups (Table 6(a)). In the HER2-negative group (*n* = 2,727, 219 events) patients receiving only AHT (HR = 2.33, 95%-CI 1.40–3.87, *P* = 0.001) or only CHT (HR = 3.15, 95%-CI 1.61–6.16, *P* = 0.001) or no adjuvant therapy at all (HR = 4.91, 95% CI 2.81–8.59, *P* < 0.001) showed a highly significant decrease in OS compared to patients with both CHT and AHT (reference group) (Table 6(b)).

## 4. Discussion

National oncological guidelines are essential for optimal treatment of cancer patients. In Germany, the majority of breast cancer patients are treated in specialized breast cancer centers which have to follow current guidelines for diagnosis and treatment of breast cancer [32, 35]. Determination of HER2 status is required in all patients with invasive breast cancer according to national [32] and international guidelines [36]. In HER2-positive early breast cancer, adjuvant CHT in combination with trastuzumab is indicated. Using data from a high-quality population-based regional cancer registry we were able to analyze guideline concordant patient care.

The steady increase in HER2 determination from 2000 (16.3%) up to 2012 (99.6%) illustrates the implementation of guidelines in routine clinical practice. The first German interdisciplinary S3 Guidelines for Diagnosis, Treatment and Follow-up Care of Breast Cancer were published in 2004 and first updated in 2008 [31]. Concerning HER2 status, the number of HER2-positive tumors declined over time. In 2000, 30.0% of tumors were HER2 positive, whereas over the years, the percentage of HER2-positive tumors continuously decreased to 12.5% in 2012. The decrease of HER2 positivity might be due to false positivity because of short fixation time and revised cutoff definition in CISH and FISH in course of the investigated period [33, 37].

All common histopathological parameters showed highly statistically significant differences between HER2-negative and HER2-positive patients as shown in previous studies [38, 39]. For instance, HER2 overexpression has been found to correlate with several adverse prognostic parameters such as large tumor size, high grade, and steroid hormone receptor negativity [39, 40].

As expected, up to 2004, trastuzumab therapy was hardly administered in primary therapy. In Europe, trastuzumab was approved for adjuvant therapy in 2006. Consequently, since 2006, the number of HER2-positive patients receiving trastuzumab continuously increased from 38.2% to 66.2% in 2011. In a cohort study from the National Comprehensive Cancer Network (NCCN) overall 44% of HER2-positive breast cancer patients received neoadjuvant or adjuvant trastuzumab with increasing proportions over time (8% of patients diagnosed in 2000, 66% of patients diagnosed in 2005, and 77% of patients diagnosed in 2007) [25]. Another study that investigated the management of HER2-positive breast cancer patients in routine clinical setting reported a rate of 54.5% of patients receiving adjuvant trastuzumab [26].

Besides analyzing the fact of guideline conform diagnostics and therapies, we demonstrated the consequences of guideline adherence on survival of patients. Guideline-conform treatment led to similar OS of HER2-positive patients compared to HER2-negative patients. This has also been shown in previous studies [27, 41, 42]. A retrospective population-based analysis of adjuvant trastuzumab use among 703 HER2-positive Canadian women, of whom 480 (68%) received trastuzumab, demonstrated highly favorable outcomes at a 2-year follow-up [28]. In patients receiving trastuzumab, the 2-year DFS was 96.1% (95% CI: 93.6% to 97.7%) and the OS was 99.3% (95% CI 97.9% to 99.8%). Among node-negative and node-positive patients, the 2-year DFS was 97.8% and 94.8% (*P* = 0.09) for the trastuzumab-treated group and 90.9% and 77.3% (*P* = 0.01) for the group not receiving trastuzumab (*n* = 223) [28]. Similar to our results, their population had better survival outcomes than those reported in the large adjuvant clinical trials. So far, only a few healthcare research studies have analyzed the impact of guideline-adherent therapies on clinical outcomes in breast cancer patients [43–45]. Overall, several prior studies confirmed that there appears to be a strong association between guideline-adherent treatment and improved survival [45–48]. Nevertheless, a study from the Netherlands Cancer Registry showed that adherence to treatment guidelines was affected by age at diagnosis but was not associated with OS in either age group [49]. Remarkably, a considerable number of patients did not receive guideline concordant therapy both in our study and previous studies, which demonstrates the difficulty of implementing guideline recommendations in routine clinical care. The reasons for this are multifactorial. Indicated therapy either never had been started or was discontinued prematurely [50]. A potential explanation for noninitiation is the preference of patients who are not willing to receive indicated therapy from the start or discontinue in the course of treatment [51]. In particular older patients may be less willing to trade current quality of life for survival [52]. Nevertheless, an observational retrospective multicenter Italian study showed that trastuzumab treatment was feasible and well tolerated in routine clinical practice [29].

Moreover, there might be a percentage of patients with small HER2-positive tumors with no CHT indication. The large international, adjuvant, randomized clinical trials have demonstrated significant improvements in DFS and OS with trastuzumab-based CHT in node-positive and/or greater than 1 cm HER2-positive tumors [14, 15, 53, 54]. Node-negative patients with smaller tumors were generally excluded [55]. Current guidelines from the NCCN recommend trastuzumab-based CHT for women with node-positive breast cancer, and women with node-negative tumors that are ≥0.6 cm.

Several other factors were assumed to influence guideline adherence in breast cancer patients in prior studies such as education, access to medical resources, health care services themselves, and an urban versus rural location [56]. However, these effects could not be replicated in the present analyses since these variables are not part of the data set.

A main cause for nonadherence to guideline recommendations may be the existence of comorbidities of which elderly patients are more often affected than younger ones. Comorbidities, especially cardiovascular diseases, may also be the cause of reduction of OS. These data have to be included in a systematic way in the update of documentation of cancer registries and will then allow systematic analyses. A retrospective study on diagnosis and treatment according to national guidelines in the Netherlands demonstrated that deviation from guidelines in elderly breast cancer patients mainly occurs due to a deliberate adjustment to patients' comorbidity and preference. Similar to our results cardiovascular disease was the most frequently observed comorbidity (53% versus 58% in our study) [44]. Moreover, a 5-year multicenter cohort study of 3,976 patients by Wöckel et al. also concluded that advanced age at initial diagnosis was associated with a reduction in guideline adherence [43]. An observational study from Schwentner et al. found lower guideline adherence in triple-negative breast cancer (TNBC) patients compared to non-triple-negative subtypes. These lower rates of guideline adherence were observed in all age groups and were most pronounced in the >65-year-old subgroup (<50 (20.9% versus 42.0%), 50–64 (25.1% versus 51.1%), and >65 (38.4% versus 74.6%)). 22.9% of their TNBC subgroup did not receive any CHT which is comparable to our results [51]. Thus, several studies have shown that patients with comorbidities are less likely to be treated according to guidelines than patients without comorbidities [52, 57, 58].

## 5. Conclusions

In conclusion, this population-based study was able to demonstrate that guideline adherent use of adjuvant trastuzumab improves OS for patients with HER2-positive breast cancer treated in routine clinical care. This extends our knowledge on the efficacy of such treatment. However, a considerable proportion of HER2-positive postmenopausal breast cancer patients did not get the appropriate therapy. It is therefore mandatory to analyze the reasons for nonadherence and to develop means to improve guideline adherence.

## Figures and Tables

**Table 1:** Time dependent rates of HER2 analyses.

Year of diagnosis	Number of patients (*N*)	HER2 status unknown (*N*, %)	HER2 status analyzed (*N*, %)	HER2 negative (*N*, %)	HER2 positive (*N*, %)
2000	430	360 (83.7%)	70 (16.3%)	49 (70.0%)	21 (30.0%)
2001	456	137 (30.0%)	319 (70.0%)	236 (74.0%)	83 (26.0%)
2002	455	72 (15.8%)	383 (84.2%)	304 (79.4%)	79 (20.6%)
2003	518	68 (13.1%)	450 (86.9%)	351 (78.0%)	99 (22.0%)
2004	568	52 (9.2%)	516 (90.8%)	421 (81.6%)	95 (18.4%)
2005	569	34 (6.0%)	535 (94.0%)	438 (81.9%)	97 (18.1%)
2006	527	8 (1.5%)	519 (98.5%)	409 (78.8%)	110 (21.2%)
2007	570	34 (6.0%)	536 (94.0%)	439 (81.9%)	97 (18.1%)
2008	560	47 (8.4%)	513 (91.6%)	423 (82.5%)	90 (17.5%)
2009	666	5 (0.8%)	661 (99.2%)	544 (82.3%)	117 (17.7%)
2010	589	6 (1.0%)	583 (99.0%)	469 (80.4%)	114 (19.6%)
2011	543	2 (0.4%)	541 (99.6%)	476 (88.0%)	65 (12.0%)
2012	540	2 (0.4%)	538 (99.6%)	471 (87.5%)	67 (12.5%)

Total	6991	827 (11.8%)	6164 (88.2%)	5030 (81.6%)	1134 (18.4%)

**Table 2:** Rate of adjuvant trastuzumab therapy in HER2-positive patients.

Year of diagnosis	Total number of HER2-positive patients (*N*)	Trastuzumab received		
		Total	Premenopausal	Postmenopausal
2000	21	0/21 (0%)	0/10 (0%)	0/11 (0%)
2001	83	1/83 (1%)	0/19 (0%)	1/64 (2%)
2002	79	2/79 (3%)	2/22 (9%)	0/57 (0%)
2003	99	2/99 (2%)	1/28 (4%)	1/71 (1%)
2004	95	2/95 (2%)	0/26 (0%)	2/69 (3%)
2005	97	34/97 (35%)	11/23 (48%)	23/74 (31%)
2006	110	42/110 (38%)	18/31 (58%)	24/79 (30%)
2007	97	57/97 (59%)	15/19 (79%)	42/78 (54%)
2008	90	53/90 (59%)	15/19 (79%)	38/71 (54%)
2009	117	64/117 (55%)	21/33 (64%)	43/84 (51%)
2010	114	65/114 (57%)	19/29 (66%)	46/85 (54%)
2011	65	43/65 (66%)	20/22 (91%)	23/43 (54%)
2012	67	24/67 (36%)	11/24 (46%)	13/43 (30%)

Total	1134	389/1134 (34%)	133/305 (44%)	256/829 (31%)

**Table 3:** Concomitant diseases in HER2-positive patients without adjuvant trastuzumab treatment.

	Premenopausal	Postmenopausal	All patients
Concomitant diseases, *n* (%)	**35 (14.8%)**	**202 (85.2%)**	**237 (100.0%)**
Cardiopulmonary	12/35 (34.3%)	126/202 (62.4%)	138/237 (58.2%)
Gastrointestinal/hepatic/renal	—	10/202 (5.0%)	10/237 (4.2%)
Metabolic	7/35 (20.0%)	22/202 (10.9%)	29/237 (12.2%)
Mental	6/35 (17.1%)	15/202 (7.4%)	21/237 (8.9%)
Others	10/35 (28.6%)	29/202 (14.4%)	39/237 (16.5%)

**Table 4:** Different systemic therapies in HER2-positive patients.

HER2-positive patients (year of diagnosis: 2006–2012)							
	Trastuzumab + CHT + AHT	Trastuzumab + CHT	CHT + AHT	CHT	AHT	No adjuvant therapy	Total
2006	27 (24.8%)	14 (12.8%)	9 (8.3%)	9 (8.3%)	40 (36.7%)	10 (9.2%)	109 (100%)
2007	30 (32.6%)	22 (23.9%)	6 (6.5%)	1 (1.1%)	21 (22.8%)	12 (13.0%)	92 (100%)
2008	31 (34.8%)	21 (23.6%)	6 (6.7%)	4 (4.5%)	16 (18.0%)	11 (12.4%)	89 (100%)
2009	45 (40.2%)	14 (12.5%)	9 (8.0%)	4 (3.6%)	24 (21.4%)	16 (14.3%)	112 (100%)
2010	44 (39.3%)	19 (17.0%)	9 (8.0%)	3 (2.7%)	18 (16.1%)	19 (17.0%)	112 (100%)
2011	29 (45.3%)	13 (20.3%)	4 (6.2%)	3 (4.7%)	10 (15.6%)	5 (7.8%)	64 (100%)
2012	15 (22.4%)	9 (13.4%)	1 (1.5%)	9 (13.4%)	9 (13.4%)	24 (35.8%)	67 (100%)

Total	221 (34.3%)	112 (17.4%)	44 (6.8%)	33 (5.1%)	138 (21.4%)	97 (15.0%)	645 (100%)

**Table 5:** Overall survival rates categorized by HER2 status and adjuvant therapy.

	3-year OS	5-year OS	6-year OS	7-year OS
HER2 positive				
Trastuzumab + CHT+ AHT	97%	96%	96%	96%
CHT + AHT	98%	98%	92%	92%
Trastuzumab + CHT	95%	92%	92%	86%
CHT	82%	65%	65%	65%
AHT	91%	83%	79%	79%
No adjuvant therapy	75%	63%	55%	55%
HER2 negative				
CHT + AHT	98%	95%	93%	92%
CHT	89%	86%	83%	83%
AHT	95%	90%	88%	88%
No adjuvant therapy	81%	75%	73%	64%

**Table:** (a) Multivariable Cox regression models in HER2-positive patients since 2006

Characteristic	Overall survival (*n* = 516, events = 53)		
	HR	95% CI	*P* value
Trastuzumab + CHT + AHT (*n* = 180, events = 4)	Reference	—	—
CHT + AHT (*n* = 35, events = 2)	2.34	0.43; 13.38	0.32
Trastuzumab + CHT (*n* = 91, events = 6)	3.80	0.87; 16.59	0.08
AHT (*n* = 106, events = 17)	4.28	1.30; 14.05	**0.017**
CHT (*n* = 24, events = 3)	9.50	1.90; 47.43	**0.006**
No adjuvant therapy (*n* = 80, events = 21)	10.44	3.02; 36.06	**<0.001**

Model is controlled for age, Ki67 categories, tumor size, nodal status, grading, and receptor status; HR: hazard ratio; 95% CI: 95% confidence interval.

**Table:** (b) Multivariable Cox regression model in HER2-negative patients since 2006

Characteristic	Overall survival (*n* = 2727, events = 219)		
	HR	95% CI	*P* value
CHT + AHT (*n* = 671, events = 23)	Reference	—	—
AHT (*n* = 1392, events = 104)	2.33	1.40; 3.87	**0.001**
CHT (*n* = 279, events = 28)	3.15	1.61; 6.16	**0.001**
No adjuvant therapy (*n* = 385, events = 64)	4.91	2.81; 8.59	**<0.001**

Model is controlled for age, Ki67 categories, tumor size, nodal status, grading, and receptor status; HR: hazard ratio; 95% CI: 95% confidence interval.
